# Chimpanzee communities differ in their inter- and intrasexual social relationships

**DOI:** 10.3758/s13420-023-00570-8

**Published:** 2023-02-01

**Authors:** Bruce S. Rawlings, Edwin J. C. van Leeuwen, Marina Davila-Ross

**Affiliations:** 1 Department of Psychology, Durham University, Durham, DH1 3LE UK; 2 Durham Cultural Evolution Research Centre, Durham University, Durham, DH1 3LE UK; 3 Centre for Comparative and Evolutionary Psychology, Psychology Department, University of Portsmouth, Portsmouth, PO1 2DY UK; 4 Animal Behaviour and Cognition, Department of Biology, University Utrecht, Utrecht, The Netherlands; 5 Department of Comparative Cultural Psychology, Max Planck Institute for Evolutionary Anthropology, Deutscher Platz 6, 04103 Leipzig, Germany

**Keywords:** Social network analysis, Chimpanzee, Male, Female, Social bonding, primate

## Abstract

Male and female human social bonding strategies are culturally shaped, in addition to being genetically rooted. Investigating nonhuman primate bonding strategies across sex groups allows researchers to assess whether, as with humans, they are shaped by the social environment or whether they are genetically predisposed. Studies of wild chimpanzees show that in some communities males have strong bonds with other males, whereas in others, females form particularly strong intrasex bonds, potentially indicative of cultural differences across populations. However, excluding genetic or ecological explanations when comparing different wild populations is difficult. Here, we applied social network analysis to examine male and female social bonds in two neighbouring semiwild chimpanzee groups of comparable ecological conditions and subspecies compositions, but that differ in demographic makeup. Results showed differences in bonding strategies across the two groups. While female–female party co-residence patterns were significantly stronger in Group 1 (which had an even distribution of males and females) than in Group 2 (which had a higher proportion of females than males), there were no such differences for male–male or male–female associations. Conversely, female–female grooming bonds were stronger in Group 2 than in Group 1. We also found that, in line with captive studies but contrasting research with wild chimpanzees, maternal kinship strongly predicted proximity and grooming patterns across the groups. Our findings suggest that, as with humans, male and female chimpanzee social bonds are influenced by the specific social group they live in, rather than predisposed sex-based bonding strategies.

**Graphical Abstract:**

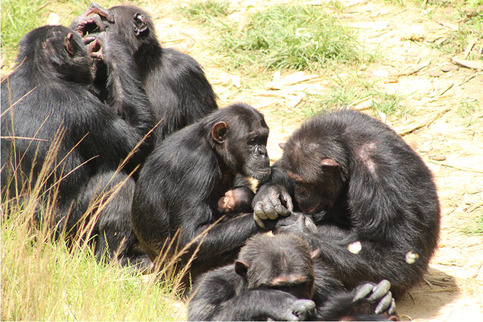

## Introduction

Human male and female social bonds and friendship networks may be culturally determined (Baumgarte, 2016; David-Barrett et al., 2015; Lu et al., 2021; Verkuyten, 1996), in addition to being biased by gender (Aukett et al., 1988; Baerveldt et al., 2004; Migliano et al., 2020; Palchykov et al., 2012; Szell & Thurner, 2013). Intragender and intergender-based bonding serves adaptive functions, but their perceived value varies across cultures (Lu et al., 2021; Quinlan, 2008; Quinlan & Quinlan, 2007). To investigate the evolutionary roots of human bonding strategies, scientists have examined sociality in nonhuman primates (Balasubramaniam et al., 2018; Benenson, 2019; Borgeaud et al., 2017; Langergraber et al., 2013; Mitani, 2009; Pasquaretta et al., 2014; Surbeck et al., 2017).

Research suggests that some nonhuman primate species exhibit differences in intrasex and intersex-based social bonding strategies across communities (Borgeaud et al., 2017; Davila-Ross et al., 2022; Stevens et al., 2007). Group differences in male and female social strategies appear to be particularly pronounced across chimpanzee populations, however. For instance, some research in Gombe National Park (Tanzania) and Kibale National Park (Uganda) shows that male–male chimpanzee social bonds are particularly strong compared with bonds among females and serve fitness benefits including to facilitate protection from other chimpanzee communities, increase status, sire offspring, boundary patrols and hunting cooperation, as well as food sharing between males (Feldblum et al., 2021; Gilby et al., 2013; Mitani, 2006, 2009; Mitani & Amsler, 2003; Mitani & Watts, 2001; Watts & Mitani, 2001), and can last over a decade (Bray & Gilby, 2020). However, other work, including those in Taï National Park (Côte d'Ivoire) and ﻿Budongo Forest (Uganda), show that females can also be highly social—especially with other females, forming long-term bonds—and display varying sociality across communities (Lehmann & Boesch, 2009;Newton-Fisher, 2006 ; Wakefield, 2013). Bonds among females may provide protection from male aggression and from dominance competition within communities (Newton-Fisher, 2006; Wakefield, 2013).

Whether nonhuman primates who live in highly similar ecological environments show group-level variation in intrasex and intersex boding strategies remains unclear. Research of this kind would shed light on the extent to which they are shaped by the social environment, in ways similar to humans, rather than being explained by ecological or genetic factors. We therefore examined the influence of the social group on male and female social bonding behaviours by comparing chimpanzees of two social groups at Chimfunshi Wildlife Orphanage, Zambia. The two groups live in highly similar naturalistic environments and they are comparable in their subspecies composition (Rawlings et al., 2014; van Leeuwen et al., 2012; van Leeuwen et al., 2018), meaning that ecological or genetic factors are unlikely to explain any cross-group differences in intra and inter sex bonding strategies.

Previous research investigating male and female chimpanzee social bonding behaviours have generally focussed on single communities. In one exception, when assessing long-term association patterns across five wild populations which differed in group sizes, sex ratio, and general demographic makeup, chimpanzees’ predominantly associated with same-sex partners (Surbeck et al., 2017). These findings are in line with other studies of single populations. For example, the male Ngogo chimpanzees (who have a high proportion of females) display close male associative bonds (Mitani & Amsler, 2003) and more frequent and successful cooperative behaviours (Mitani & Watts, 1999; Watts & Mitani, 2001).

Male presence is also suggested to reduce female aggression towards immigrating females, and males intervene in female–female aggression (Kahlenberg, Thompson, Muller, & Wrangham, 2008b). Social network analysis has also shown that as the Taï community group size decreased over time, females become more central to their group, ostensibly as competition and threat of aggression decreased (Lehmann & Boesch, 2009). However, the latter study only examined female sociality, meaning the role males played in such changes is unclear. Finally, other work suggests that social constraints and demographics, including group size, immigration of new group members, and differences in age and rank impact chimpanzee social behaviours and bonding patterns, particularly alliance formations (Kahlenberg, Thompson, & Wrangham, 2008a; Mitani, 2006; Mitani et al., 2002). In sum, while these studies hint that chimpanzee bonding behaviours differ across populations, ruling out ecological or genetic explanations remains difficult when comparing different communities in the wild.

For a comprehensive assessment of social bonding, we applied social network analysis (SNA). SNA allows scientists to measure social group structures and is a robust quantitative approach for constructing group social relationships at group and individual levels (Puga-Gonzalez et al., 2019). SNA has been previously applied to describe social relationships of several primate, species including humans (Dufour et al., 2011; Gradassi et al., 2022; Migliano et al., 2020; Pasquaretta et al., 2014; Puga-Gonzalez et al., 2019; Salali et al., 2016; Schel et al., 2013; van Leeuwen et al., 2018). We collected social network data based on proximity and grooming, which are widely used predictor of chimpanzee bonds (Díaz et al., 2020; Kanngiesser et al., 2011; Roberts & Roberts, 2016a; Schel et al., 2013; van Leeuwen et al., 2018; Wakefield, 2013). However, some studies have reported that proximity and grooming networks differentially predict other social behaviours, such as successful transmission of information (Hasenjager et al., 2021; Hoppitt, 2017; van Leeuwen et al., 2020). Thus, including both measures allowed us to examine whether they similarly or differentially predicted male and female bonding strategies across the study groups. It also allowed some comparisons with human social network studies, which use proximity and communication to measure association patterns (Guo et al., 2015; Migliano et al., 2020; Page et al., 2017; Van Cleemput, 2012).

In addition, we examined the potential impact of kinship and age on associations within and across sex groups. Maternal kinship influences chimpanzee cooperation, affiliation, and prosociality (Clark, 2011; Langergraber et al., 2009; Samuni et al., 2021) and age-related differences have been shown to affect chimpanzee proximity and social behaviours (Benenson, 2019; Kawanaka, 1989; Mitani et al., 2002). Previous studies at Chimfunshi Wildlife Orphanage (CWO) have reported substantial group differences in chimpanzees’ grooming behaviours (van Leeuwen et al., 2012), extractive foraging techniques (Rawlings et al., 2014), play vocalizations (Davila-Ross et al., 2011), and social dynamics more generally (van Leeuwen et al., 2018). The four main study groups at CWO show consistent differences in attributes of their sociality (e.g., co-feeding tolerance), with corresponding effects on behaviours known to affect fitness (van Leeuwen et al., 2021). As such, we conducted our study testing the hypothesis that the two largest groups of chimpanzees at CWO differed in their sex-specific sociality.

## Methods

**Subjects, study site, and data collection** Subjects were 61 chimpanzees housed in two groups at Chimfunshi Wildlife Orphanage (CWO), Zambia. Group 1 comprised 22 subjects: 11 males (mean age = 18.22, *SD* = 11.14) and 11 females (mean age = 17.82, *SD* = 9.70), Group 2 comprised 39 subjects: 10 males (mean age = 13.06 years, *SD* = 7.93) and 29 females (mean age = 17.59, *SD* = 8.38), see Table 1 for group demographics. Chimpanzees under 4 years of age were not considered in this study as their location and behaviour was strongly contingent on their mothers’.

**Table 1:** Number of subjects by age and sex for Groups 1 and 2

	Group 1	Group 2
Adolescent and adult males (8+ years)	8	6
Adolescent and adult females (8+ years)	8	24
Juvenile males (4–8 years)	3	4
Juvenile females (4–8 years)	3	5
Infants* (under 4 years)	3	8

* Infants were not considered in this study

The chimpanzees of Group 1 live in a 65-hectare enclosure and Group 2 chimpanzees in a 72-hectare enclosure. The two enclosures are approximately 200 meters apart, formed of the Miombo Woodland, providing large, naturalistic environments which are separated by fencing. Data were collected in 2013 (July–September), between the hours of 06:30–18:00. For more details of the CWO chimpanzees and their environment, see (Cronin et al., 2014; Forrester et al., 2015; Rawlings et al., 2014; Van Leeuwen et al., 2012; van Leeuwen et al., 2018).

Proximity data were collected through focal sampling individuals for 5 minutes and recording all individuals within 10 meters of the focal subject. Following Cronin et al. (2014) and Whitehead (2008), we took a 1/0 sampling per day approach to maximize data independence (i.e., if two individuals were observed associating once or more on the same day they were scored 1, and if not this dyad scored 0). Focal order was randomized before each day of data collection, providing a balance between morning and afternoon data for individuals. There was a total of 460 focals for Group 1 (mean per individual = 20.91, *SD* = 2.76), and 845 focals for Group 2 (mean per individual = 20.12, *SD* = 0.53). We also constructed sociograms for both groups to visualize their respective network structures. For proximity data, we distinguished party co-residence (proximity to focal <10 meters) and direct association (proximity to focal <1 meter). For grooming data, we recorded each time a focal individual was involved in a grooming bout (either giving or receiving).

**Association measures** To assess social bond strength, association matrices based on the simple-ratio index were calculated. The simple-ratio index is calculated as follows:

1 $$\frac{x}{x+{Y}_{AB}+{Y}_A+{Y}_B},$$

where x is the number of sampling periods A and B were observed associated; Y_A_, represents the number of sampling periods with just A identified; Y_b_, represents the number of sampling periods with just B identified; and Y_ab_, is the number of sampling periods with A and B identified but not associated (Whitehead, 2008). As noted above, to optimize data independency, the sampling period was set to “date” (i.e., 24hrs). The association index score for each dyad is between 0 and 1 (0 = never observed together; 1 = always observed together). In Group 1, the number of dyads examined was *N* = 55, 121, and 55 for male–male, male–female, and female–female dyads, respectively. In Group 2, the number of dyads compared was *N* = 45, 208, and 488 for male–male, male–female, and female–female dyads (FF, FM, MM), respectively.

**Statistical analysis** General linear mixed models (GLMM) were used to examine whether the two study groups of chimpanzees differed in the relationship between dyad sex type (FF, FM, MM) and association index (simple ratio association [SRA]; Hoppitt & Farine, 2018) while including maternal kinship and age difference between dyads as covariates. Specifically, we ran three GLMMs (Baayen, 2008) in the R statistical environment (Version 4.1.2; R Core Team, 2020). First, we modelled SRA based on party co-residence (i.e., proximity to focal <10 meters) with beta error distribution and logit link function. Second, we modelled SRA based on direct association (i.e., proximity to focal <1 meter). Given that more than half of the resulting SRAs were 0, here, we applied a hurdle approach where we first modelled yes/no association with binomial error structure and logit link function, and subsequently the nonzero associations (henceforth ‘magnitude’) with beta error distribution and logit link function. Third, we modelled SRA based on grooming associations (i.e., comprising both grooming given and received by and from the focal). Here, for the same reason, we applied the same hurdle approach. For kinship, we identified all individuals that were maternally related (binary coded – yes/no), such that a mother and an offspring, and maternal siblings would be coded as maternally related (grandmothers, ‘aunts,’ and ‘uncles’ were not). For Group 1, 17/231 dyads were maternally related, and for Group 2, 35/741 dyads were maternally related. Age differences between dyads were calculated in years and months apart. The main fixed variable was dyad sex type in interaction with group, whereas maternal relatedness and age difference were entered as covariates. To account for non-independence of the response variable owing to repeated observations, we included both the focal and partner (together making up the dyad) as random intercept variables. We applied a standard regression method capable of accounting for repeated measures of individuals as well as controlling for influential variables, while assessing the strength of the predicted variables on the response (Baayen, 2008; Bolker et al., 2009). Given that we worked with observational data collected on different groups, with inherent biases regarding the selecting and therefore the assessment of certain individuals (e.g., less neophobic individuals, or individuals with high gregariousness; Farine & Whitehead, 2015; Whitehead, 2008), we additionally treated the inputted datastream (i.e., the data used as response for the GLMMs) in order to minimize the influence of such biases on the inferential framework (Farine & Aplin, 2019; van Leeuwen et al., 2019). This treatment has been proposed to benefit from permutations before the data are condensed into network indices (Bejder et al., 1998), hence the name “prenetwork” or “datastream” permutations (Farine, 2017). The preferred relationships (based on the different input measures) are computed following standard social network methods (i.e., association indices; Whitehead, 2008), where we chose to use the currently most supported form of “simple-ratio” indices (Hoppitt & Farine, 2018). However, given that we were interested in which social and demographic variables determined these indices, we furthermore regressed them onto our variables of interest, specifically dyad sex combinations.

In order to obtain unbiased *p* values for the central question of whether the two groups of chimpanzees differed in the extent to which the dyad sex types associated, we applied data-stream (aka prenetwork) permutations (*n* = 1,000; Farine, 2013) in which we randomly assigned associations across the group members in a given day, while retaining the original frequency of associations per given day. The generated random networks were each analyzed with the same GLMMs as the original data (see above). We applied a model comparison between a full model including the interaction between dyad sex type and group, and a reduced model without the respective interaction yet with the main effects retained (Dobson & Barnett, 2018). For each iteration, we extracted the deviance difference between the models and compared these with the deviance difference of the original models (i.e., sum(Δdeviance ≤ Δdeviance_random_)/1000) to obtain a *p* value for the respective interaction (henceforth “P_rand_”). This approach was chosen to acknowledge the bias in observation effort due to certain focal subjects being more likely to be observed than others (e.g., owing to differences in enclosure usage). GLMMs were run using the R packages *lme4* (Bates et al., 2015) and *glmmTMB* (Brooks et al., 2017). Separate dyad sex contrasts were analyzed with the *emmeans* package (Lenth, 2020).

Sociograms were generated using the R package *igraph* (Csárdi & Nepusz, 2006). The generated sociograms depict the simple ratio association indices where the nodes represent individuals (red = females; blue = males) and the edges represent the dyadic tie-strength based on the association data. Networks were laid out using the Fruchterman–Reingold weighted algorithm, which increases the uniformity of edge-length and minimizes edge crossings. The graphs display communities generated by the spinglass algorithm (Reichardt & Bornholdt, 2006).

## Results

**Fig. 1:**
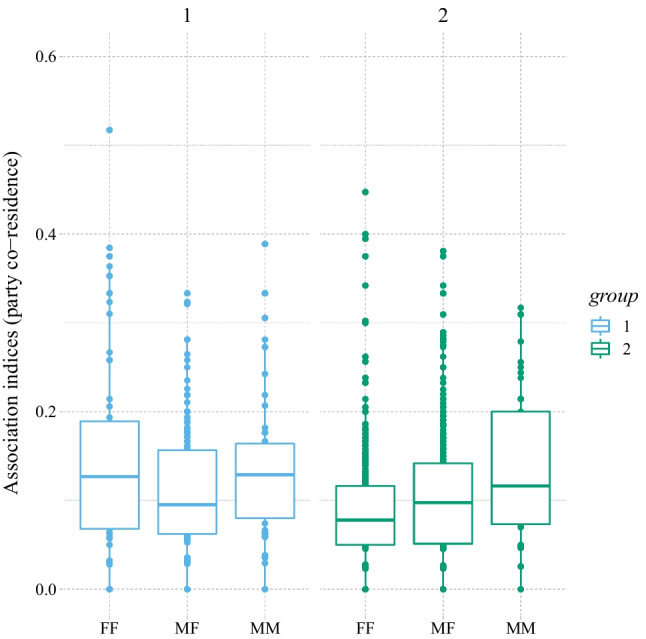
Party co-residence (within 10 meters) for the different dyad sex types separately for Group 1 (blue; left) and Group 2 (green, right). Dots represent dyadic association scores, the boxes represent the interquartile range (IQR); the vertical lines attached to the boxes represent Q1 − 1.5 IQR (lower) and Q3 + 1.5 IQR (upper); medians are represented by the bold, horizontal lines within the boxes. (Color figure online)

**Fig. 2:**
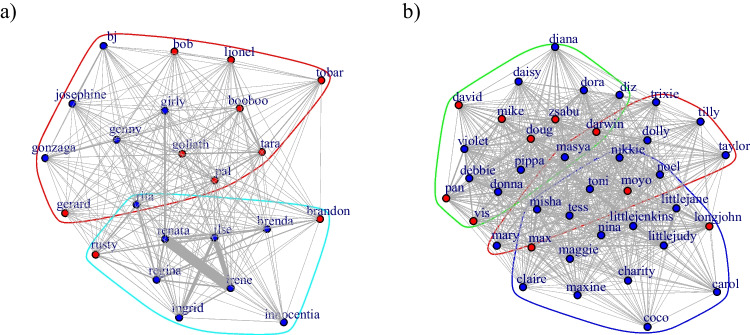
Simple ratio association indices based on party co-residence data (within 10 meters distance) for (**a**) Group 1 and (**b**) Group 2. The nodes represent individuals (red = females; blue = males), the edges represent the dyadic tie strengths. The first letter of individuals’ names follow maternal lines, and as such, reflect maternal kinship. (Color figure online)

**Party co-residence:** The two groups of chimpanzees differed significantly in their party compositions in terms of sex combinations (likelihood ratio test [LRT]: χ^2^ = 16.87, *df* = 2, P_rand_ < 0.001; see Fig. 1). Specifically, association strengths of FF combinations were more pronounced in Group 1 (mean ± *SD* = 0.17 ± 0.17) than in Group 2 (0.08 ± 0.06; *t* value = 2.38, *p* = .018), while there was no such difference for the MF (*t* = −0.71, *p* = .476) and MM dyads (*t* = 0.71, *p* = .478; see Fig. 1 and for the respective sociograms, see Fig. 2). Maternal relatedness was highly predictive of co-residence association strength (LRT: χ^2^ = 90.17, *df* = 1, *p* < .001; estimate ± *SE* = 0.96 ± 0.09). Age difference did not affect party co-residence (LRT: χ^2^ = 1.79, *df* = 1, *p* = .18).

**Fig. 3:**
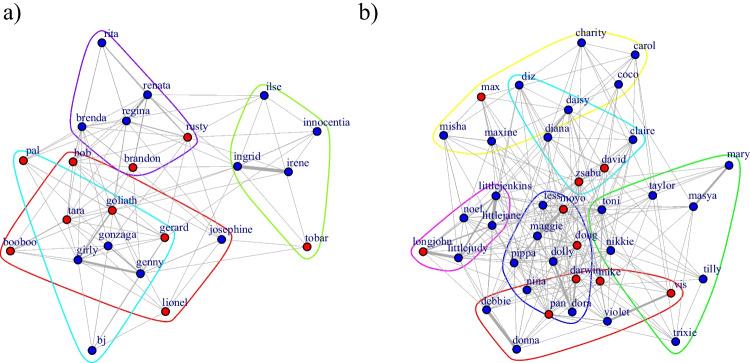
Simple ratio association indices based on 1m proximity data for (**a**) Group 1 and (**b**) Group 2. The nodes represent individuals (red = females; blue = males), the edges represent the dyadic tie strengths. (Color figure online)

**Proximity** (A) *Probability* of having established a proximity association (yes/no). The two groups of chimpanzees did not differ in their probabilities to be in proximity as a function of dyadic sex combinations (LRT: χ^2^ = 3.14, *df* = 2, *p* = .247; see Fig. 3). Maternal relatedness was highly predictive of the probability of having established a proximity association (LRT: χ^2^ = 86.30, *df* = 1, *p* < .001; estimate ± *SE* = 3.02 ± 0.39). Age difference did not obviously affect proximity probability (LRT: χ^2^ = 2.40, *df* = 1, *p* = .12).

(B) *Magnitude* of proximity within established association dyads (association >0). The two groups did also not differ in the extent to which established proximity dyads were in proximity as a function of dyadic sex combination (LRT: χ^2^ = 3.39, *df* = 2, *P*_rand_ = 0.168; Fig. 3). Again, maternal relatedness was highly predictive of the magnitude of proximity (LRT: χ^2^ = 130.70, *df* = 1, *p* < .001; estimate ± *SE* = 0.99 ± 0.08). Age difference did not obviously affect magnitude of proximity, although a trend was detected such that a larger age difference predicted a higher magnitude of proximity (LRT: χ^2^ = 2.86, *df* = 1, *p* = .09; estimate ± *SE* = 0.05 ± 0.03).

**Fig. 4:**
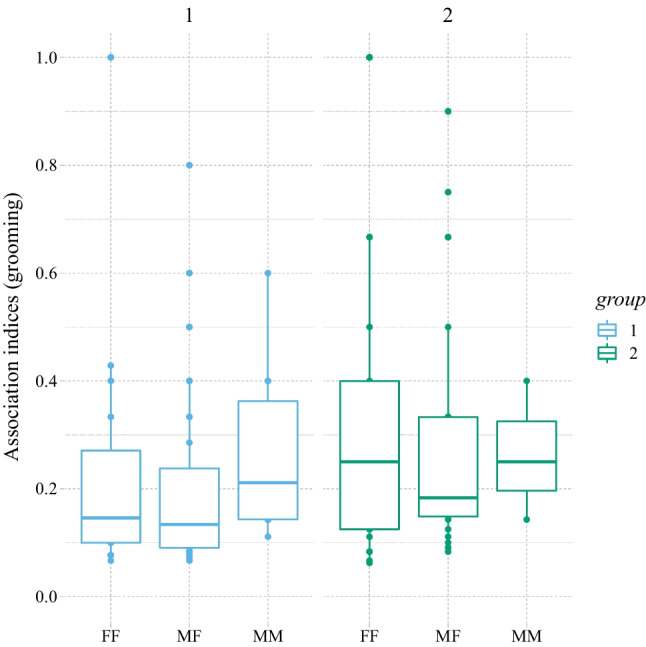
Magnitudes of grooming associations (including only established grooming bonds) for the different dyad sex types separately for Group 1 (blue; left) and Group 2 (green, right). Dots represent dyadic association scores, the boxes represent the interquartile range (IQR); the vertical lines attached to the boxes represent Q1 − 1.5 IQR (lower) and Q3 + 1.5 IQR (upper); medians are represented by the bold, horizontal lines within the boxes. (Color figure online)

**Fig. 5:**
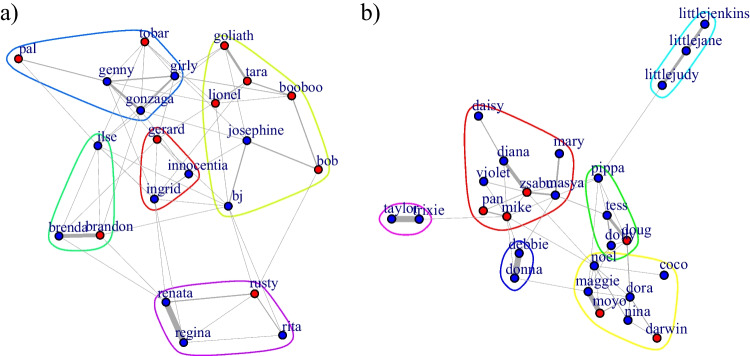
Simple ratio association indices based on grooming data for (**a**) Group 1 and (**b**) Group 2. The nodes represent individuals (red = females; blue = males), the edges represent the dyadic tie-strengths. (Color figure online)

**Grooming** (A) *Probability* of having established a grooming association (yes/no). The two groups of chimpanzees did not differ in the probability of grooming as a function of dyadic sex combination (LRT: χ^2^ = 2.97, *df* = 2, *p* = .28). Maternal relatedness was highly predictive of the probability of grooming (LRT: χ^2^ = 40.85, *df* = 1, *p* < .001; estimate ± *SE* = 2.50 ± 0.43). Age difference did not obviously affect grooming probability (LRT: χ^2^ = 0.03, *df* = 1, *p* = .86). (B) *Magnitude* of grooming in established bonds (association >0). The two groups did significantly differ in the extent to which established grooming dyads engaged in grooming as a function of dyadic sex combination (*P*_rand_ < 0.001; Figs. 4 and 5) with the most pronounced group differences in grooming magnitude between FF dyads (estimate ± *SE* = −0.631 ± 0.356), followed by the MF dyads (estimate ± *SE* = −0.519 ± 0.320) and the MM dyads (estimate ± *SE* = 0.184 ± 0.633; see Figs. 4 and 5). Again, maternal relatedness was highly predictive of the magnitude of grooming (LRT: χ^2^ = 37.14, *df* = 1, *p* < .001; estimate ± *SE* = 1.54 ± 0.24). Age difference did not obviously affect grooming magnitude (LRT: χ^2^ = 0, *df* = 1, *p* = .97).

## Discussion

To better understand how group demographics impact sex differences in chimpanzee sociality, we provide an in-depth analysis on male and female social bonding in populations that share ecological conditions and do not genetically differ. The results showed that the dyad types bonded differently across the two chimpanzee groups, both in terms of patterns of party co-residence and grooming patterns. While female–female proximity associations were significantly stronger in Group 1 (which had an even distribution of males and females) than Group 2 (which had a higher proportion of females than males), there were no such group differences for male–male or male–female associations. Conversely, female–female grooming bonds were stronger in Group 2 than Group 1. These group differences cannot be explained by ecological and genetic influences, as the groups live in similar ecological environments and are comparable in their subspecies composition (Rawlings et al., 2014; van Leeuwen et al., 2012; van Leeuwen et al., 2018). Thus, we provide robust evidence that the social bonding of chimpanzees is shaped differently depending on the social group they live in. In turn, these results progress the debate regarding whether nonhuman primates show sex-specific, or more flexible, bonding behaviours (Bray & Gilby, 2020; Mitani, 2006, 2009; Surbeck et al., 2017; Wakefield, 2013) by directly comparing two groups of neighbouring chimpanzees in the same study rather than carrying out indirect comparisons or comparisons of communities who live in different locations.

Distal proximity (within 10 m) and grooming are different forms of bonding, potentially serving different functions, while both contributing to social cohesion. In chimpanzees, grooming between dyads has been associated with reduced aggression (Schel et al., 2013), coalition forming, postconflict resolution and agnostic support (Muller & Mitani, 2005; Schel et al., 2013), and has been argued to be an especially strong indicator of social bonding (Fedurek & Dunbar, 2009; Roberts & Roberts, 2016b). It is thus plausible that in Group 2, which was larger and had a high proportion of females, female–female dyadic grooming may serve to minimize intrasex aggression and competition and facilitation stronger bonds. Indeed, in the Ngogo chimpanzees, which also has a high proportion of females compared with males, females form comparatively strong association bonds and cluster together (Wakefield, 2013).

The findings that female–female showed stronger proximity associations in Group 1 than Group 2 may reflect a different strategy by females in this group. Previous research has shown that chimpanzees’ distal proximity does not predict grooming patterns, which was suggested to reflect that grooming reflects more targeted, richer bonding strategies while distal proximity allows individuals to maintain a larger set of social relationships (Roberts & Roberts, 2016b). Thus, in Group 1, which was smaller and had a higher concentration of males, it is possible that the females used proximity to maintain relationships with most or all other females in the group, whereas females in the larger Group 2 used grooming to form particularly strong bonds with targeted other females. This in turn may suggest that different bonding strategies are differentially optimal in different social environments. Indeed, studies of social transmission have reported that proximity and grooming networks differentially predict the spread of information, where one is highly predictive of social transmission and the other is less so, or not at all (Hasenjager et al., 2021; Hoppitt, 2017; van Leeuwen et al., 2020). Future work could investigate how demographics, including female estrous cycles (Surbeck et al., 2021), may impact the function of social behaviours such as proximity and grooming, and in turn, the expression of group-specific bonding dynamics.

Maternal kinship was a strong predictor of both proximity and grooming patterns. This contrasts work with wild chimpanzees where females disperse from their communities. For example in the Ngogo chimpanzees, most female social bonds were outside of kinship lines (Langergraber et al., 2009) and kinship did not meaningfully impact male affiliation or cooperation patterns (Langergraber et al., 2007). Likewise, kinship did not predict reciprocal grooming in the Tai chimpanzees (Gomes et al., 2009). However, studies with captive chimpanzees appear to show stronger proximity bonds and grooming associations along kinship lines (Clark, 2011; Díaz et al., 2020; Kanngiesser et al., 2011). It is possible in environments such as zoos and sanctuaries (like CWO) where there is no dispersal, mothers and their offspring form strong bonds into adulthood, and, in turn, provide social support during conflicts or in cooperative contexts (Clark, 2011).

Previously, researchers have discussed the role of group demographics on male and female social bonding based on indirectly comparing results drawn from one community in Africa, such as Gombe National Park or Kibale National Park to data reported from other communities such as Budongo Forest or Taï National Park (Langergraber et al., 2009; Lehmann & Boesch, 2004; Mitani & Amsler, 2003; Newton-Fisher, 2006; Wakefield, 2013; Watts & Mitani, 2001), or by comparing different communities across Africa (Surbeck et al., 2017). However, in such cases, ruling out factors such as ecological and genetic variation, among other explanations, remains difficult. Based on our findings involving chimpanzee groups in shared ecological environments and with comparable genetic composition, we conclude that male and female social bonds that may be shaped by the social environment, in line with previous work on the CWO chimpanzees (Rawlings et al., 2014; Van Leeuwen et al., 2012; van Leeuwen et al., 2014, 2018, 2019). It is important to note however, that other factors we have not considered here such as levels of within-group aggression and personality types (Massen & Koski, 2014; Rawlings et al., 2020), or polymorphic variation in receptor genes that are related to the expression of social behaviour in chimpanzees (Staes et al., 2014) may also impact bonding in chimpanzees. Future research could investigate how such variables influence associations within and between sex groups in these semi-wild groups as well as other chimpanzee communities.

In addition, it is important to consider to what extent methodological differences across studies may impact results on social relationships. For example, treatment of proximity measures differs between studies. Here, party co-residence was calculated as proximity focal <10 meters. While some studies in the wild have also used this approach (Roberts et al., 2019), others have differed—using, for example, within 50 m (Langergraber et al., 2013; Rushmore et al., 2013) or simply within visual range of the focal individual (Wakefield, 2013). Likewise, as here, some studies have used focal follow protocols (Langergraber et al., 2013; Lehmann & Boesch, 2004; Rushmore et al., 2013; Schel et al., 2013; Wakefield, 2013), while others have also included group scan sampling to collect proximity data (Funkhouser et al., 2018). Although data from these approaches are correlated, group scan sampling has been shown to be slightly less accurate in predicting chimpanzee foraging behaviour (Gilby et al., 2010). It is thus important to consider the methodological approaches taken when comparing across studies, and whether this may impact results. Further, although the chimpanzees at CWO live in large, naturalistic environments, systematic comparisons between sanctuary living chimpanzees and wild communities are needed to examine whether, and how, living environment impacts bonding strategies.

In conclusion, we examined the social bonding strategies of sanctuary-living chimpanzees that are comparable in ecological and subspecies composition. Our findings on these strategies also add to an already large body of work showing that the CWO chimpanzees exhibit group differences in a range of domains including extractive foraging, play vocalizations, co-feeding tolerance, prosociality, and grooming behaviours. We conclude that male and female chimpanzee social bonding strategies at least in part shaped by social factors, possibly culturally, in ways comparable to humans. Social bonding has played an essential role in human evolution, facilitating cooperation and maintaining cohesion in expanding group sizes, and our results shed light on how the social environment influences intra- and intersex/gender-based sociality.

## Data Availability

The data used in this study are available at: https://datadryad.org/stash/share/oCFayXGSG3rKzkfFUVAet10_HHHvy4tSDdMlNCnt1X8
